# Theoretical Analysis of the Mechanism of Fracture Network Propagation with Stimulated Reservoir Volume (SRV) Fracturing in Tight Oil Reservoirs

**DOI:** 10.1371/journal.pone.0125319

**Published:** 2015-05-12

**Authors:** Yuliang Su, Long Ren, Fankun Meng, Chen Xu, Wendong Wang

**Affiliations:** School of Petroleum Engineering, China University of Petroleum (East China), Qingdao, Shandong, P. R. China; University of Zaragoza, SPAIN

## Abstract

Stimulated reservoir volume (SRV) fracturing in tight oil reservoirs often induces complex fracture-network growth, which has a fundamentally different formation mechanism from traditional planar bi-winged fracturing. To reveal the mechanism of fracture network propagation, this paper employs a modified displacement discontinuity method (DDM), mechanical mechanism analysis and initiation and propagation criteria for the theoretical model of fracture network propagation and its derivation. A reasonable solution of the theoretical model for a tight oil reservoir is obtained and verified by a numerical discrete method. Through theoretical calculation and computer programming, the variation rules of formation stress fields, hydraulic fracture propagation patterns (FPP) and branch fracture propagation angles and pressures are analyzed. The results show that during the process of fracture propagation, the initial orientation of the principal stress deflects, and the stress fields at the fracture tips change dramatically in the region surrounding the fracture. Whether the ideal fracture network can be produced depends on the geological conditions and on the engineering treatments. This study has both theoretical significance and practical application value by contributing to a better understanding of fracture network propagation mechanisms in unconventional oil/gas reservoirs and to the improvement of the science and design efficiency of reservoir fracturing.

## Introduction

Stimulated reservoir volume (SRV) fracturing has become an important technique to improve well production and the recovery of unconventional reservoirs in oil/gas field development. With the development of the SRV fracturing technique, studying the mechanism of fracture network propagation with SRV fracturing has been an academic focus, including the variation rules of stress fields, mechanical mechanisms of fracture propagations and the corresponding geological and engineering factors.

Fracture propagation is strongly influenced by the mechanical interaction between neighboring fractures throughout the fracture growth history. This mechanical interaction, called the stress shadowing effect, is quantified using a boundary-element numerical model originally developed for natural-fracture propagation simulation. Erdogan and Sih [1] calculated the stress distribution near the crack tip under two special conditions, which involving symmetric and skew-symmetric case, by using stress field equations of the composite type I-II fracture at fracture tips in polar coordinates. In a 2D, plane-strain, displacement discontinuity solution, Crouch and Starfield [2] described the normal and shear stresses acting on one fracture element caused by the opening and shearing displacement discontinuities from all fracture elements. To consider the 3D effect due to finite fracture height, Olson [3] introduced a 3D correction factor to the influence coefficients. Since then, many scholars have done a lot of work in this regard, whose including Olson [4], Weng [5] and Kresse [6] and [7], et al. Nagel et al. [8] presented a discrete element model numerical study of multi-well completions simulated in a fully hydro-mechanical coupled fashion, and considered the influence of changes in the in-situ pressure in order to more completely understand the mechanical interactions between propagating hydraulic fractures and natural fractures during multi-well completions.

The methods of microseismic monitoring, numerical simulation and rock physics experiments have typically been used to analyze the mechanisms of fracture propagation patterns (FPP) with SRV fracturing in unconventional reservoirs. Complex hydraulic fracture geometry is regarded as a common place occurrence in naturally fractured reservoirs largely based on the interpretation of microseismic data by Fisher et al. [9], Maxwell et al. [10], Daniels et al. [11] and Le Calvez et al. [12]. In recent years, some progress has been made in this study by use of the numerical simulation method with flexible ways, which including Planar Fracture Model (PFM) and Unconventional Fracture Model (UFM) involving Complex Fracture Network Model (CFNM) and Discrete Fracture Network Model (DFNM). Zhang et al. [13] presented a PFM incorporating a full solution of coupled elasticity and fluid equations, but it is limited to the 2D plane strain conditions. Olson et al. [14] and [15] presented and modified the CFNM that is capable of predicting hydraulic fracture propagation and interaction with pre-existing natural fractures based on the Extended Finite Element Method (XFEM), and it was developed by Weng et al. [5] and Kresse et al. [7]. However, great uncertainty exists with respect to the nature of the natural fracture system and importantly how the hydraulic fracture interacts with it. To provide a framework to address these uncertainties, DFNM was presented by Rogers et al. [16] and was improved based on the discrete element models by Nagel et al. [17]. Laboratory experiments are another way to simulate the process of hydraulic fracture propagation in per-existing fracture formation. Blanton [18], Renshaw and Pollard [19] and Gu et al. [20] studied and expanded the approach of hydraulic fracture crossing or arrest in frictional interfaces within Devonian shale and hydro-stone. Several events may occur during the time period of the hydraulic fracture propagating towards the natural fracture and beyond. Zhou et al. [21] delineated natural fractures by inserting paper sheets of varying strength into cement blocks to simulate per-existing fractures of various “shear strength”. Olson et al. [22] performed hydro-stone block experiments to examine the effect of cemented natural fractures on hydraulic fracture propagation. The results indicate that oblique embedded fractures are more likely to divert a fluid-driven hydraulic fracture and hydraulic fracture and natural fracture interaction took three usual patterns.

Griffith [23] first postulated the maximum stress theory, which can predict the direction of crack growth. After its initial publication, the theory was re-examined by Erdogan and Sih [1], who proposed that the maximum circumferential stress in the fracture tip should be regarded as a controllable parameter of the composite crack extension by experimenting with the extension of a central crack under symmetrical plane loading in brittle materials (Plexiglass). Blanton [18] and [24] used blocks of Devonian Shale cast in hydro-stone to show that a hydraulic fracture crosses a pre-existing fracture only under high differential stresses and high angles of approach. He noted that in most cases, fractures were either diverted or arrested by pre-existing fractures. Warpinski and Teufel [25] recognized the differences between in-situ stresses as the governing factor of fracture cross-over or fracture diversion in mine-back experiments and presented the corresponding fracture propagation criterion. The resulting effect of the discontinuities depends mainly on ancillary parameters (permeability of the joints, frictional properties, in-situ stresses, etc.). Based on this former research, a simple criterion for predicting whether a fracture will propagate across a frictional interface oriented perpendicular to the approaching fracture has been derived by Renshaw and Pollard [19]. However, this criterion has many restrictions on intersections. Hopkins et al. [26] analyzed the performance of hydraulic fracturing in fractured Antrim shale and concluded that complex fracture paths are primarily controlled by the geometry of the natural fractures. This conclusion contrasted with Blanton’s theory, which limits natural fracture dilation to locations with low differential stresses. Consequently, a simplified multiple hydraulic fracture propagation model was presented by Olson [14] to demonstrate several important in situ and operational procedure parameters that influence the resultant fracture network geometry from horizontal wells and in naturally fractured formations. The geometry is strongly influenced by horizontal plane in situ stress contrasts, net pressure magnitude, hydraulic fracture height and the presence of natural fractures. Subsequently, Olson and Dahi-Taleghani [4] defined a parameter called the relative net pressure and developed a simplified numerical model to account for the mechanical interaction between pressurized fractures. The results show that fracture pattern complexity is strongly controlled by the magnitude of the hydraulic fracture net pressure relative to the in situ horizontal differential stress and the geometry of the natural fractures.

Previous studies have investigated theoretical models based on the crack mechanics theory or rock stress experiments, but all the above approaches may be considered to be less rigorous because the branch-fracture propagation description is not comprehensive. The branch-fracture propagation description assumes the stress field and propagation angle to be constant. In fact, the interference of the combined stress field would result in a continuous, mutative propagation angle at the fracture tip. This study presents a theoretical model of fracture network propagation with SRV fracturing in tight oil reservoirs. Based on the modified DDM, mechanical mechanism analysis and initiation and propagation criteria, a reasonable solution of the theoretical model is obtained and verified with the numerical discrete method. Using theoretical arithmetic and computer programming, the variation in the formation of the stress field, FPP and branch fracture propagation angle and pressure are analyzed.

## Geological Conditions of Fracture Network Formation in Tight Oil Reservoirs

SRV fracturing is essential in the development of tight oil reservoirs, and the formation of fracture networks is key in increasing production. Several important geological factors affect the formation of fracture networks: rock mineral composition, rock mechanical properties, natural fractures and horizontal stress field.

1. Mineral Composition. Mineral composition is key to rock brittleness [27]. The brittleness of a mineral increases when it contains silicon or calcium, making the mineral more easily fractured and facilitating the activation of natural fractures. Therefore, an induced fracture network is easily formed after fracturing. However, high clay concentrations may create a softer and more ductile formation, complicating the formation of stable flow paths and complex fracture networks.
2. Rock Mechanical Properties. To a large extent, rock mechanical properties are affected by the mineral composition (or rock brittleness). The concept of rock brittleness combines both Poisson’s ratio and Young’s modulus. Poisson’s ratio is used to reflect the ability of the rock to fail under stress; as values of Poisson’s ratio decrease, the brittleness of the rock increases. Young’s modulus reflects the ability of the rock to maintain a fracture [28]. A higher Young’s modulus indicates more brittle rock.
3. Natural Fractures. The orientation of the natural fracture will affect the propagation of the hydraulic fracture and the formation of fracture network [29]. The results show that when the angle between the natural fracture and hydraulic fracture is less than 30 degrees, the probability that the natural fracture will be activated and the original propagation path will be changed increases, causing the easier formation of a fracture network. However, once the angle is larger than 60 degrees, the formation of a fracture network becomes impossible. In the case that the angle is between 30 and 60 degrees, the formation of a fracture network depends on the horizontal stress difference.
4. Horizontal Stress Field. Under the condition of a low horizontal stress difference, the hydraulic fracture would propagate along natural fractures, and a radial fracture network would be induced. Hence, the stress anisotropy in fractured formations with low horizontal stress difference is weak, and as fractures propagate along different directions, the resulting net pressure difference is less [30] and [31]. Therefore, hydraulic fractures propagate more easily along natural fractures in random directions to form a fracture network.

## Theoretical Model of Fracture Network Propagation

### 3.1 Combined Stress Field Distribution

In view of the problems of stress concentration in the fracture tips, this section uses a modified displacement discontinuity method (DDM) by introducing the shape function as a Lagrange interpolation cardinal function to improve the precision of discontinuous displacement for the discrete state. The computational model of the combined stress field is constructed by the stress shadow effect and stress boundary condition. This model considers the influence of the fracture length on the stress field distribution during the fracturing treatments.

#### 3.1.1 DDM Based on the Lagrange Interpolation

DDM is based on the analytical solution to the problem of constant discontinuity in displacement over a finite line segment in the *x*, *y* plane of an infinite elastic solid. The basic solutions proposed by DDM to the problem of conventional boundary and crack boundary are discontinuous displacements [2].

**Fig 1:**
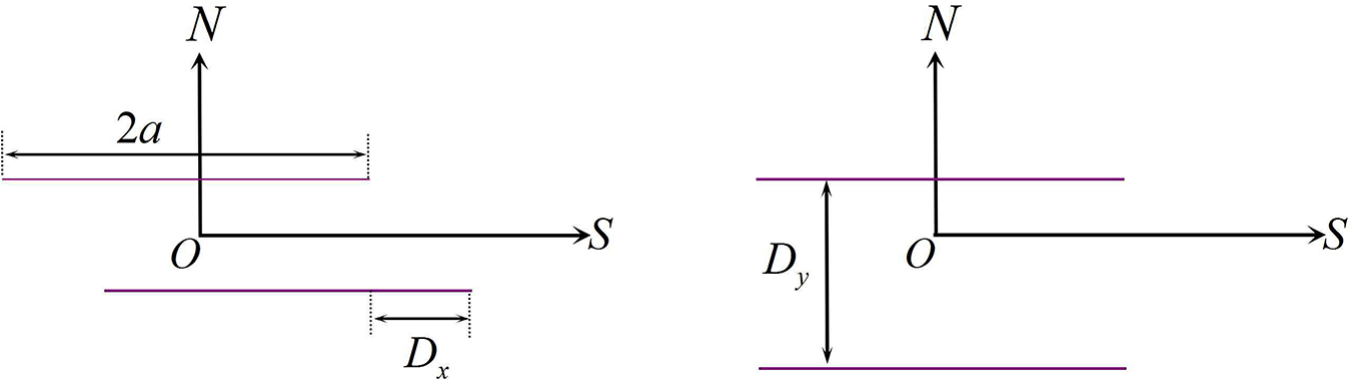
2-D displacement discontinuity fracture element for a discrete state.

Considering a 2-D model, when the surfaces of several arbitrary fracture elements bear a stress field at a discrete state, slippage will occur between the upper and lower surfaces. This slippage is displacement discontinuity, and the relative magnitude of the slippage is a discontinuous displacement. We divide the single fracture into several equal elements, each of which has a length of 2*a*. *SON* is the local coordinate system. *D*_*s*_ and *D*_*n*_ are the tangential and normal discontinuous displacements, respectively (Fig 1). When |*s*| ≤ *a*, the equation of a discontinuous displacement can be defined as follows:

$$\{\begin{array}{c} D_{s}=u_{s}(s,0_{-})-u_{s}(s,0_{+})\\ D_{n}=u_{n}(n,0_{-})-u_{n}(n,0_{+}) \end{array}$$ (1)

where *u*_*s*_ and *u*_*n*_ are the tangential and normal displacement components, respectively; ‘+’ indicates the upper surface and ‘-’ indicates the lower surface of the fracture element.

To improve the accuracy of the discontinuous displacement between one fracture element and the other two adjacent elements, the shape function *N*_*m*_ (*ξ*)(*m* = 1 ∼ 3) is introduced as the Lagrange-interpolation cardinal function. Consequently, the discontinuous displacement of arbitrary point can be rewritten as follows:

$$\{\begin{array}{c} D_{s}(\xi )=\sum_{m=1}^{3}N_{m}(\xi ){\left(D_{s}\right)}_{m}\\ D_{n}(\xi )=\sum_{m=1}^{3}N_{m}(\xi ){\left(D_{n}\right)}_{m} \end{array}$$ (2)

where $N_{m}(\xi )=\frac{\prod_{k=1}^{3}\left(\xi -\xi_{k}\right)}{\prod_{k=1}^{3}\left(\xi_{m}-\xi_{k}\right)}$; (*k* ≠ *m*) is the shape function; and *ξ*_1_ = −2*a*, *ξ*_2_ = 0, *ξ*_3_ = 2*a*. Therefore, $N_{1}(\xi )=\frac{\xi^{2}-2a\xi }{8a^{2}}$, $N_{2}(\xi )=\frac{\xi^{2}-4a^{2}}{4a^{2}}$, and $N_{3}(\xi )=\frac{\xi^{2}+2a\xi }{8a^{2}}$.

Based on the theory of elastic mechanics, assuming that the fracture element surface produces symmetrical tangential and normal discontinuous displacements, the relational expression among the displacements (*u*_*s*_ and *u*_*n*_), stress fields (*σ*_*ss*_, *σ*_*nn*_ and *τ*_*sn*_) and discontinuous displacements (*D*_*s*_ and *D*_*n*_) of the fracture element in the local coordinate system are given by the following:

$$\{\begin{array}{l} u_{s}^{i}=\sum_{m=1}^{3}{\left(D_{s}\right)}_{m}[2(1-\nu )\bar{F_{3}}-n\bar{F_{5}}]+\sum_{m=1}^{3}{\left(D_{n}\right)}_{m}[-(1-2\nu )\bar{F_{2}}-n\bar{F_{4}}]\\ u_{n}^{i}=\sum_{m=1}^{3}{\left(D_{s}\right)}_{m}[(1-2\nu )\bar{F_{2}}-n\bar{F_{4}}]+\sum_{m=1}^{3}{\left(D_{n}\right)}_{m}[2(1-\nu )\bar{F_{3}}+n\bar{F_{5}}]\\ \sigma_{ss}^{i}=2G\sum_{m=1}^{3}{\left(D_{s}\right)}_{m}(2\bar{F_{4}}+n\bar{F_{6}})+2G\sum_{m=1}^{3}{\left(D_{n}\right)}_{m}(-\bar{F_{5}}+n\bar{F_{7}})\\ \sigma_{nn}^{i}=2G\sum_{m=1}^{3}{\left(D_{s}\right)}_{m}(-n\bar{F_{6}})+2G\sum_{m=1}^{3}{\left(D_{n}\right)}_{m}(-\bar{F_{5}}-n\bar{F_{7}})\\ \tau_{sn}^{i}=2G\sum_{m=1}^{3}{\left(D_{s}\right)}_{m}(-\bar{F_{5}}+n\bar{F_{7}})+2G\sum_{m=1}^{3}{\left(D_{n}\right)}_{m}(-n\bar{F_{6}}) \end{array}$$ (3)

where $\bar{F_{n}}(n=2∼5)$ is the derivative function of *f*_*m*_(*s*, *n*) that is only related to the fracture element coordinates; $\bar{F_{2}}=f_{m,s}=\frac{\partial f_{m}(s,n)}{\partial s}$, $\bar{F_{3}}=f_{m,n}=\frac{\partial f_{m}(s,n)}{\partial n}$, $\bar{F_{4}}=f_{m,sn}=\frac{\partial^{2}f_{m}(s,n)}{\partial s\partial n}$, $\bar{F_{5}}=f_{m,ss}=-f_{m,nn}=\frac{\partial^{2}f_{m}(s,n)}{\partial s^{2}}$, $\bar{F_{6}}=f_{m,snn}=\frac{\partial^{3}f_{m}(s,n)}{\partial s\partial n^{2}}$, $\bar{F_{7}}=f_{m,nnn}=\frac{\partial^{3}f_{m}(s,n)}{\partial n^{3}}$, where $f_{m}(s,n)=-\frac{1}{4\pi (1-\nu )}\int_{-a}^{a}N_{m}(\xi )\text{ln}{\left[{\left(s-\xi \right)}^{2}+n^{2}\right]}^{\frac{1}{2}}d\xi$.

For the function $F_{m}(I_{0},I_{1},I_{2})=\int_{-a}^{a}N_{m}(\xi )\text{ln}{\left[{\left(s-\xi \right)}^{2}+n^{2}\right]}^{\frac{1}{2}}d\xi$, where $I_{i}=\int_{-a}^{a}\xi^{i}\;\text{ln}{\left[{\left(s-\xi \right)}^{2}+n^{2}\right]}^{\frac{1}{2}}d\xi$ (*i* = 0~2), the analytical formula can be given as follows:

$$I_{0}=n(\theta_{1}-\theta_{2})-(s-a)\text{ln}\;r_{1}+(s+a)\text{ln}\;r_{2}-2a$$

$$I_{1}=sn(\theta_{1}-\theta_{2})+\frac{1}{2}(n^{2}-s^{2}+a^{2})(\text{ln}\;r_{1}-\text{ln}\;r_{2})-as$$

$$I_{2}=\frac{n}{3}(3s^{2}-n^{2})(\theta_{1}-\theta_{2})+\frac{1}{3}(3sn^{2}-s^{3}+a^{2})\text{ln}\;r_{1}-\frac{1}{3}(3sn^{2}-s^{3}-a^{3})\text{ln}\;r_{2}-\frac{2}{3}a(s^{2}-n^{2}+\frac{a^{2}}{3})$$

where $\theta_{1}=\text{arctan}\frac{n}{s-a}$ and $\theta_{2}=\text{arctan}\frac{n}{s+a}$ are the angles between the S-axis and two lines connecting any point (*s*, *n*) to (-*a*, 0) and (*a*, 0), respectively; $r_{1}=\sqrt{{\left(s-a\right)}^{2}+n^{2}}$ and $r_{2}=\sqrt{{\left(s+a\right)}^{2}+n^{2}}$ are the distances of any point (*s*, *n*) to (-*a*, 0) and (*a*, 0), respectively.

#### 3.1.2 Combined Stress Field and Stress Boundary Conditions

In the local coordinate system *SO’N*, the stress field (normal stress and shear stress) of the fracture element *i* under an in-situ stress field can be defined as follows:

$$\{\begin{array}{l} {\left(\sigma_{nn}^{i}\right)}_{0}={\left(\sigma_{xx}^{i}\right)}_{0}{\;\text{sin}}^{2}\;\beta^{i}+{\left(\sigma_{yy}^{i}\right)}_{0}\;{\text{cos}}^{2}\;\beta^{i}-{\left(\tau_{xy}^{i}\right)}_{0}\;\text{sin}\;2\beta^{i}\\ {\left(\tau_{sn}^{i}\right)}_{0}=\frac{{\left(\sigma_{yy}^{i}\right)}_{0}-{\left(\sigma_{xx}^{i}\right)}_{0}}{2}\text{sin}\;2\beta^{i}+{\left(\tau_{xy}^{i}\right)}_{0}\;\text{cos}\;2\beta^{i} \end{array}$$ (4)

where ${\left(\sigma_{nn}^{i}\right)}_{0}$ is the normal stress of fracture element *i* in the N direction; ${\left(\tau_{sn}^{i}\right)}_{0}$ is the shear stress of fracture element *i*; ${\left(\sigma_{xx}^{i}\right)}_{0}$ and ${\left(\sigma_{yy}^{i}\right)}_{0}$ are the original normal stresses of fracture element *i* in the X and Y directions, respectively; and ${\left(\tau_{xy}^{i}\right)}_{0}$ is the original shear stress of fracture element *i*.

**Fig 2:**
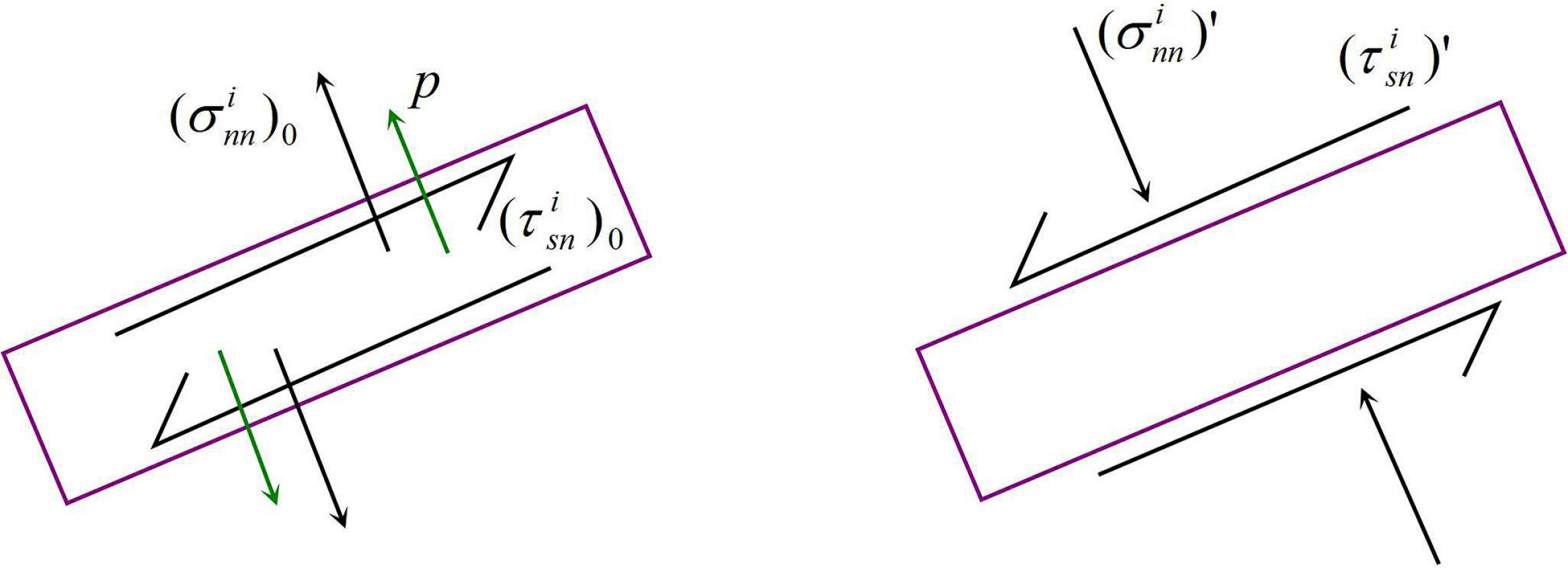
Stress distribution of the fracture element with the stress shadow effect.

For a fracture element, assuming the surface displays a symmetrical net pressure, the normal and shear stresses on the surface of the element are generated from the superposition of the stress shadows produced by other elements (as shown in Fig 2). Combining the equations of the additional stress field [2], the stress field (normal and shear stress) of fracture element *i* becomes the following:

$$\{\begin{array}{l} -{\left(\tau_{sn}^{i}\right)}_{0}=(\tau_{sn}^{i})'=\sum_{j=1}^{N}\sum_{m=1}^{3}C_{ss}^{ij}D_{s}^{j}+\sum_{j=1}^{N}\sum_{m=1}^{3}C_{sn}^{ij}D_{n}^{j}\\ -p-{\left(\sigma_{nn}^{i}\right)}_{0}=(\sigma_{nn}^{i})'=\sum_{j=1}^{N}\sum_{m=1}^{3}C_{ns}^{ij}D_{s}^{j}+\sum_{j=1}^{N}\sum_{m=1}^{3}C_{nn}^{ij}D_{n}^{j} \end{array}$$ (5)

where $C_{ss}^{ij}$ and $C_{sn}^{ij}$ are the elastic coefficients of the normal stress due to the effects of shear and the open discontinuous displacements ($D_{s}^{j}$ and $D_{n}^{j}$) of element *j* on fracture element *i* in the X direction, respectively; $C_{ss}^{ij}=2G[2{\text{cos}}^{2}\;\beta \bar{F_{4}}+\text{sin}2\beta \bar{F_{5}}+n(\text{cos}2\beta \bar{F_{6}}-\text{sin}2\beta \bar{F_{7}})]$, $C_{sn}^{ij}=2G[-\bar{F_{5}}+n(\text{sin}\;2\beta \bar{F_{6}}+\text{cos}\;2\beta \bar{F_{7}})]$; $C_{ns}^{ij}$ and $C_{nn}^{ij}$ are the elastic coefficients of the normal stress due to the effects of shear and the open discontinuous displacements ($D_{s}^{j}$ and $D_{n}^{j}$) of element *j* on fracture element *i* in the Y direction, respectively; $C_{ns}^{ij}=2G[2{\text{sin}}^{2}\;\beta \bar{F_{4}}-\text{sin}2\beta \bar{F_{5}}-n(\text{cos}2\beta \bar{F_{6}}-\text{sin}2\beta \bar{F_{7}})]$, $C_{nn}^{ij}=2G[-\bar{F_{5}}-n(\text{sin}\;2\beta \bar{F_{6}}+\text{cos}\;2\beta \bar{F_{7}})]$; *β* is the angle between the N direction of element *i* and the X axis.

The above equations are the stress boundary conditions for the discrete fracture element. The equations on the left are the known stress boundary conditions, and the right side are the 2N variables containing the fracture element discontinuous displacements ($D_{s}^{j}$ and $D_{n}^{j}$) which can be solved through the establishment of 2N equations.

Both the propagation of hydraulic fractures and their extension upon encountering natural fractures are influenced by the combined stress field during the SRV fracturing of naturally fractured reservoirs. The combined stress field is formed by the superposition of two stress fields, which are the in-situ stress field caused by the long-term geological conditions and the additional stress field induced by multi-fracture propagation. The equations of the combined stress field are as follows:

$$\{\begin{array}{l} \sigma_{xx}={\left(\sigma_{xx}^{i}\right)}_{0}+(\sigma_{xx}^{i})'={\left(\sigma_{xx}^{i}\right)}_{0}+\sum_{j=1}^{N}\sum_{m=1}^{3}C_{ss}^{ij}D_{s}^{j}+\sum_{j=1}^{N}\sum_{m=1}^{3}C_{sn}^{ij}D_{n}^{j}\\ \sigma_{yy}={\left(\sigma_{yy}^{i}\right)}_{0}+(\sigma_{yy}^{i})'={\left(\sigma_{yy}^{i}\right)}_{0}+\sum_{j=1}^{N}\sum_{m=1}^{3}C_{ns}^{ij}D_{s}^{j}+\sum_{j=1}^{N}\sum_{m=1}^{3}C_{nn}^{ij}D_{n}^{j}\\ \tau_{xy}={\left(\tau_{xy}^{i}\right)}_{0}+(\tau_{xy}^{i})'={\left(\tau_{xy}^{i}\right)}_{0}+\sum_{j=1}^{N}\sum_{m=1}^{3}C_{sns}^{ij}D_{s}^{j}+\sum_{j=1}^{N}\sum_{m=1}^{3}C_{snn}^{ij}D_{n}^{j} \end{array}$$ (6)

where *σ*_*xx*_ and *σ*_*yy*_ are the normal stresses in the X and Y directions, respectively, under the combined stress field; *τ*_*yy*_ is the shear stress in the combined stress field; $(\sigma_{xx}^{i})'$ and $(\sigma_{yy}^{i})'$ are the additional normal stresses of fracture element *i* in the X and Y directions, respectively; $(\tau_{xy}^{i})'$ is the additional shear stress of fracture element *i*; $C_{sns}^{ij}$ and $C_{snn}^{ij}$ are the elastic coefficients of the shear stress from the effects of the shear and open discontinuous displacements ($D_{s}^{j}$ and $D_{n}^{j}$) of fracture element *j* on fracture element *I*, respectively, $C_{sns}^{ij}=2G[\text{sin}2\beta \bar{F_{4}}-\text{cos}2\beta \bar{F_{5}}+n(\text{sin}2\beta \bar{F_{6}}+\text{cos}2\beta \bar{F_{7}})]$, $C_{snn}^{ij}=2G[-n(\text{cos}\;2\beta \bar{F_{6}}-\text{sin}\;2\beta \bar{F_{7}})]$.

#### 3.1.3 Numerical Solution by Programming

**Fig 3:**
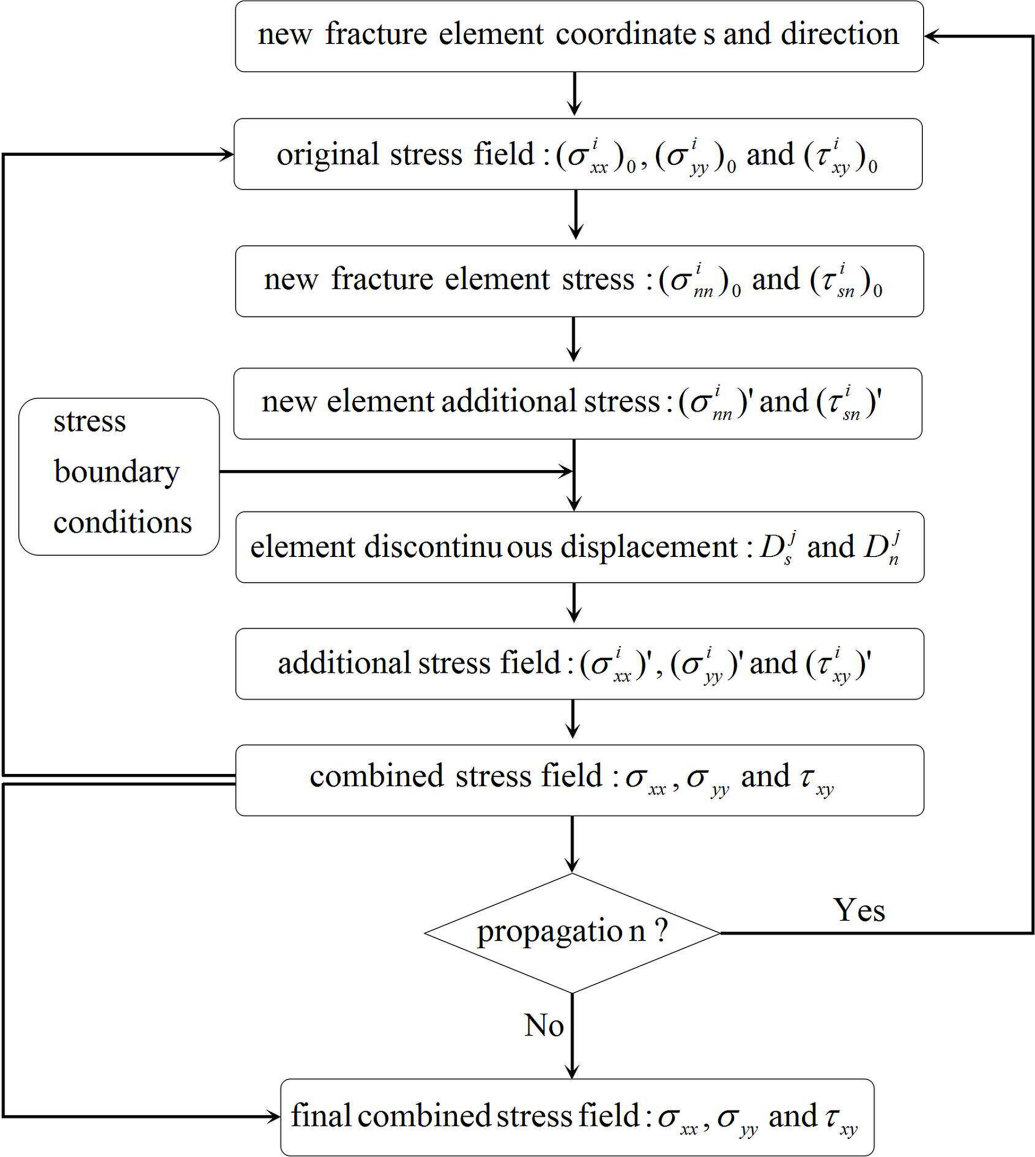
Flow chart of the solution to the combined stress field.

In the process of iteratively solving the pressure and geometric parameters for each fracture element, the additional stress field (normal and shear stress) produced by stress shadow effects must be recalculated at every time step. The stress field must be superimposed at the final time step to determine the new composite stress field distribution in the global coordinate system *XOY*. Fig 3 displays the flow chart for the solution to the combined stress field at a chosen position.

### 3.2 Mechanical Mechanism of the FPP

Hydraulic and natural fracture interactions occurred in three forms in laboratory experiments [22]: (1) hydraulic fractures that bypass natural fractures by propagating around them (via height growth, not curving), (2) hydraulic fractures arresting into natural fractures and then diverting along and sometimes kinking off the end of the natural fractures, and (3) a combination of bypass and diversion.

**Fig 4:**
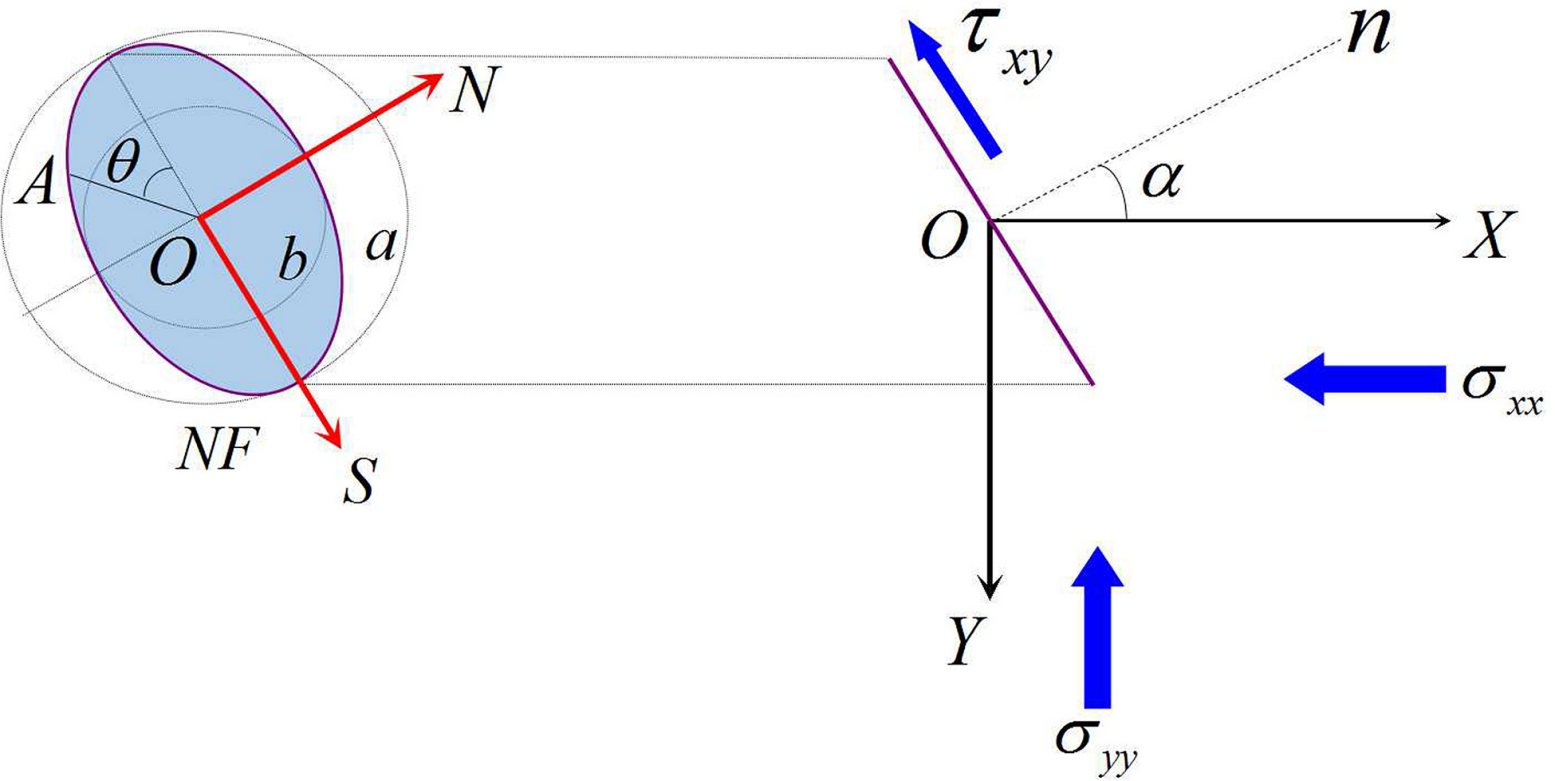
Classification of the FPP.

Because natural fractures develop in tight oil reservoirs, SRV fracturing often induces complex fracture network growth. However, to form the complex network, the natural fractures must initiate and produce open rupture or shear slippage, which can effectively link natural fractures to form branch fractures. When a hydraulic fracture propagates along a natural fracture tip after intersecting with the natural fracture, branching of the hydraulic fracture to form a complex fracture network is possible. However, the fluid pressure at the intersection point must exceed the fluid pressure drop from the intersection point of the natural fracture tip and satisfy the initiation and propagation criteria at the natural fracture tip [30]. Based on the above analysis of the mechanical relationship between the fluid pressure at the intersection point of a hydraulic and a natural fracture and other geological factors (normal stress, shear stress and rock tensile strength), the hydraulic fracture and natural fracture interaction occurred in the following three patterns (as shown in Fig 4).

#### FPP 1

The hydraulic fracture does not pass through the natural fracture but induces the diversion of fracture propagation. The propagation of the hydraulic fracture diverts when the fluid pressure of the intersection point cannot cause the hydraulic fracture to pass through the natural fracture. Rather, the hydraulic fracture induces natural fracture slippage, initiates at the tips, and diverts propagation along the direction of maximum principal stress. The mechanical condition is given by the following:

$$\sigma_{n}+\Delta p_{s}<p_{i}<\sigma_{t}+T_{0}$$ (7)

where *σ*_*n*_ and *σ*_*t*_ are the normal stresses perpendicular and parallel, respectively, to the natural fracture surface, expressed in MPa; *T*_*0*_ is the rock tensile strength, MPa; *p*_*i*_ is the fluid pressure at the intersection point, MPa; Δ*p*_*s*_ is the pressure drop between the intersection point and fracture tips, expressed in MPa.

According to the theory of two-dimensional linear elastic fracture mechanics, the following equations can be obtained: $\sigma_{n}=\frac{1}{2}(\sigma_{xx}+\sigma_{yy})-\frac{1}{2}(\sigma_{xx}-\sigma_{yy})\text{cos}2\beta$, $\sigma_{t}=\frac{1}{2}(\sigma_{xx}+\sigma_{yy})+\frac{1}{2}(\sigma_{xx}-\sigma_{yy})\text{cos}2\beta$, where *θ* is the contact angle, $\left[-\frac{\pi }{2},\frac{\pi }{2}\right]$; $\Delta p_{s}=\frac{4(p_{i}-p_{0})}{\pi }\sum_{n=0}^{\infty }\frac{1}{2n+1}\text{exp}\left[-\frac{{\left(2n+1\right)}^{2}\;\pi^{2}k_{nf}t}{4\phi_{nf}\mu C_{t}L_{nf}^{2}}\right]\text{sin}\frac{(2n+1)\pi }{2}$, where *k*_*nf*_ is the natural fracture permeability, expressed in mD; *μ* is the fluid viscosity in the reservoir, expressed in mPa.s; *ϕ*_*nf*_ is the dimensionless natural fracture porosity; *C*_*t*_ is the natural fracture comprehensive compression coefficient, expressed in 1/MPa; *p*_0_ is the original fluid pressure in the reservoir, expressed in MPa; *L*_*nf*_ is the natural fracture length, expressed in m; and *p*_*i*_ = *p*_*net*(*i*)_ + *σ*_*yy*_, where *p*_*net*(*i*)_ is the net pressure at the intersection point, expressed in MPa.

Thus, the formula for the net pressure at the intersection point becomes the following:

$$\frac{1}{2}(\sigma_{xx}-\sigma_{yy})(1+\text{cos}\;2\beta )+T_{0}>p_{net(i)}>\frac{1}{2}(\sigma_{xx}-\sigma_{yy})(1-\text{cos}\;2\beta )+\Delta p_{s}$$ (8)

#### FPP 2

he hydraulic fracture passes through natural fracture, inducing a partial opening of the natural fracture. The hydraulic fracture passes directly through the natural fracture and propagates along the initial direction (the maximum principal stress direction). According to the Blanton criteria [24], the fluid pressure at the intersection point must satisfy the following formula:

$$p_{i}>\sigma_{t}+T_{0}$$ (9)

Therefore, the following formula for the net pressure at the intersection point is obtained:

$$p_{net(i)}>\frac{1}{2}(\sigma_{xx}-\sigma_{yy})(1+\text{cos}\;2\beta )+T_{0}$$ (10)

#### FPP 3

The hydraulic fracture passes through the natural fracture, inducing the diversion of natural fracture propagation. Simultaneously, the net pressure at the intersection point cannot only exceed the rock tensile strength and normal stress to pass through the natural fracture but also must induce natural fracture slipping.

### 3.3 Fracture Initiation and Propagation Criteria

In this section, the mathematical model of the branch fracture initiation and propagation criteria is constructed based on the maximum circumferential stress theory and the stress intensity factor theory of the composite type I-II fracture.

#### 3.3.1 Maximum Circumferential Stress Theory

Based on the maximum circumferential stress criterion, we can assume that the crack extends along the direction of the circumferential stress, reaching a maximum value; the crack initiates in this direction when the circumferential stress reaches a critical value.

**Fig 5:**
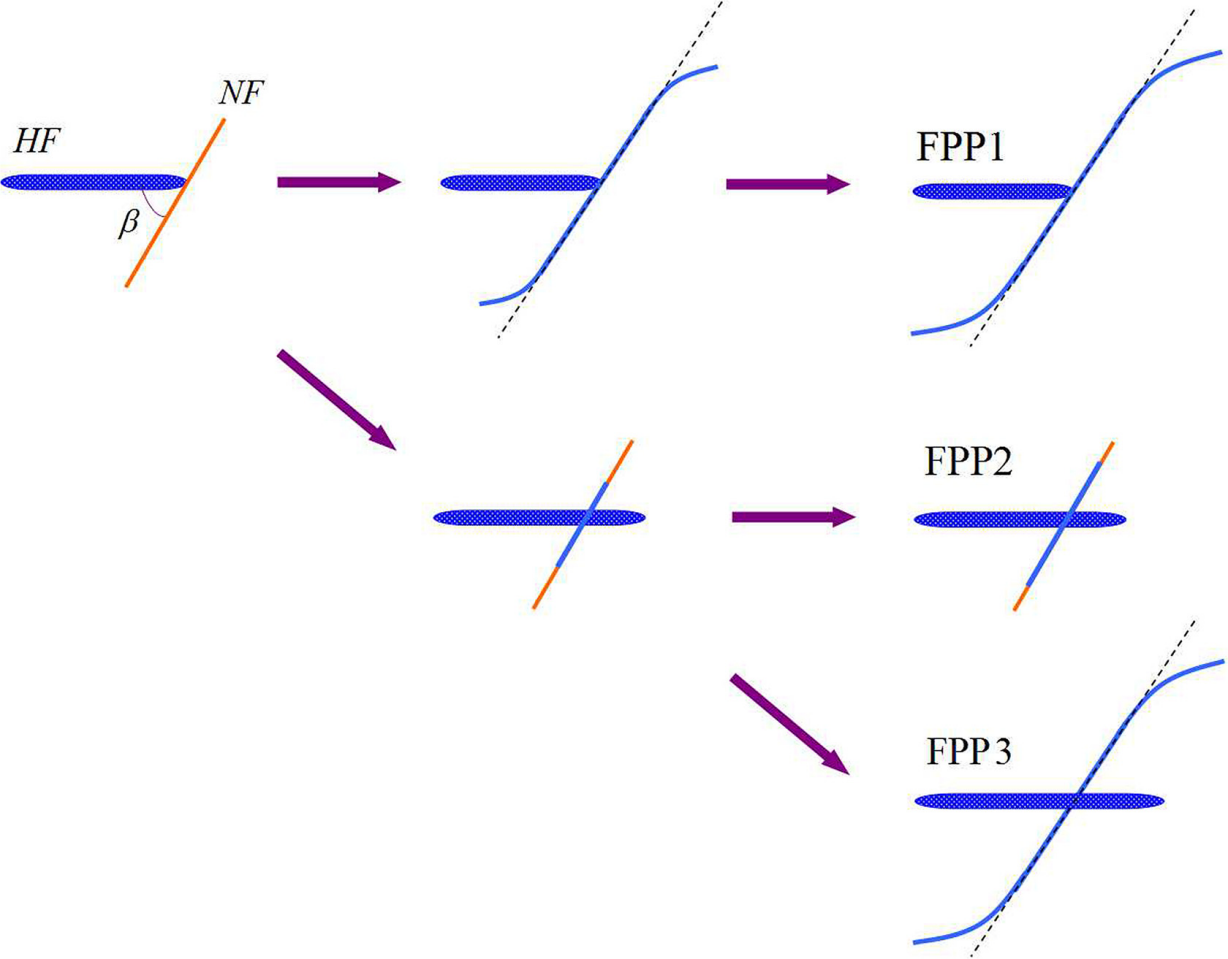
Stress and strain field at the fracture tip.

(1) The stress field equations for a composite type I-II fracture at a fracture tip in polar coordinates (as shown in Fig 5) can be written as follows:

$$\left\{ \begin{array}{l} \sigma_{rr}=\frac{1}{2\sqrt{2\pi r}}\left[K_{\text{I}}(3-\text{cos}\;\theta )\text{cos}\frac{\theta }{2}+K_{\text{II}}(3\;\text{cos}\;\theta -1)\text{sin}\frac{\theta }{2}\right]\\ \sigma_{\theta \theta }=\frac{1}{\sqrt{2\pi r}}\text{cos}\frac{\theta }{2}\left[K_{\text{I}}\;{\text{cos}}^{2}\frac{\theta }{2}-\frac{3}{2}K_{\text{II}}\;\text{sin}\;\theta \right]\\ \tau_{r\theta }=\frac{1}{2\sqrt{2\pi r}}\text{cos}\frac{\theta }{2}\left[K_{\text{I}}\;\text{sin}\;\theta +K_{\text{II}}(3\;\text{cos}\;\theta -1)\right] \end{array}\right.$$ (11)

(2) The propagation angle equations of a composite type I-II fracture (for which the circumferential stress is equal to the limiting value) can be written as follows:

$$\frac{\partial \sigma_{\theta \theta }}{\partial \theta }=0,\;\frac{\partial^{2}\sigma_{\theta \theta }}{\partial^{2}\theta }<0$$ (12)

The equation can be rewritten as follows:

$$\frac{-3}{4\sqrt{2\pi r}}\text{cos}\frac{\theta }{2}[K_{\text{I}}\;\text{sin}\;\theta +K_{\text{II}}(3\text{cos}\;\theta -1)]=0$$ (13)

Because $\text{cos}\left(\frac{\theta }{2}\right)\neq 0$, the corresponding simplified equation can be given as follows:

$$\text{cos}\;\theta_{0}=\frac{3\pm M\sqrt{8+M^{2}}}{9+M^{2}}$$ (14)

where $M=\frac{K_{\text{I}}}{K_{\text{II}}}$. Considering that the value of *θ*_*0*_ is constant when the shear stress changes, Eq (14) can be rewritten as $\text{cos}\;\theta_{0}=\frac{3+\left|M\right|\sqrt{8+M^{2}}}{9+M^{2}}$. This equation shows that the propagation angle does not depend on the material properties because the parameters in this equation do not contain the material constants *E* and *ν*.

(3) Maximum Circumferential Stress

**Fig 6:**
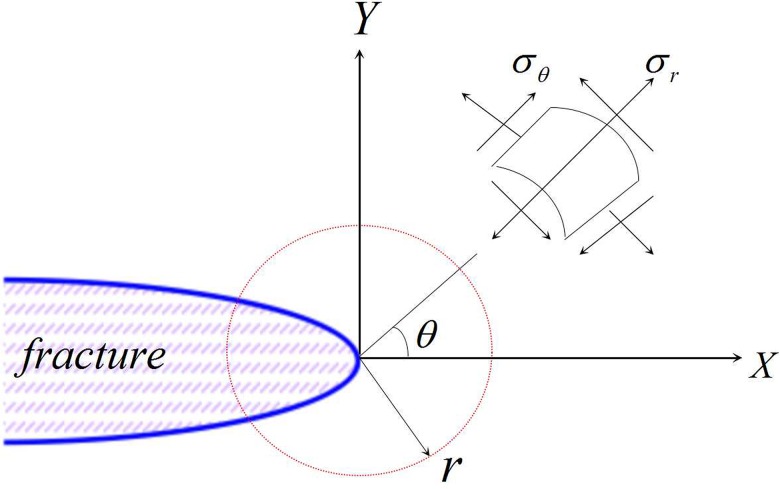
Variation curve of the *g*_*θθ*_(*θ*) function.

In the function ${\left(\sigma_{\theta \theta }\right)}_{\text{max}}=\frac{1}{\sqrt{2\pi r}}\left[K_{\text{I}}g_{\theta \theta }^{I}(\theta )-K_{\text{II}}g_{\theta \theta }^{II}(\theta )\right]$, $g_{\theta \theta }^{I}(\theta )={\text{cos}}^{3}\frac{\theta }{2}$, $g_{\theta \theta }^{II}(\theta )=-\frac{3}{2}\text{cos}\frac{\theta }{2}\text{sin}\;\theta$, and their curves are shown in Fig 6.

For the composite type I-II fracture in Fig 6, when *M* is positive and negative, the conditions at the fracture tips achieve maximum circumferential stress values of -70.53° *≤ θ ≤* 0 and 0 *≤ θ ≤* 70.53°, respectively.

(4) Fracture Initiation Criteria

$${\left(\sigma_{\theta \theta }\right)}_{\text{max}}\;(K_{I},K_{II},\theta_{0})={\left(\sigma_{\theta \theta }\right)}_{\text{c}r}$$ (15)

where (*σ*_*θθ*_)_*cr*_ is the critical value of the maximum circumferential stress and depends on the fracture toughness of the type I fracture, which is also a constant related only to the material properties and independent of the composite fracture state. The fracture criterion must describe the type I fracture, and the material properties must be invariant.

For the pure type I fracture, *θ*_*0*_ equals zero. The value of *K*_*I*_ equals *K*_*IC*_ when initiating the fracture. Therefore, the critical stress equation of fracture initiation becomes the following:

$${\left(\sigma_{\theta \theta }\right)}_{\text{c}r}=\frac{K_{\text{IC}}}{\sqrt{2\pi r}}$$ (16)

Consequently, the composite type I-II fracture initiation criteria becomes the following:

$$K_{\text{eq}}=\frac{1}{2}\text{cos}\frac{\theta_{0}}{2}\left[K_{\text{I}}(1+\text{cos}\;\theta_{0})-3K_{\text{II}}\;\text{sin}\;\theta_{0}\right]\geq K_{\text{IC}}$$ (17)

where *K*_*eq*_ and *K*_*IC*_ are the equivalent fracture strength and the fracture toughness at the fracture tips, respectively.

#### 3.3.2 Stress Intensity Factor (SIF)

**Fig 7:**
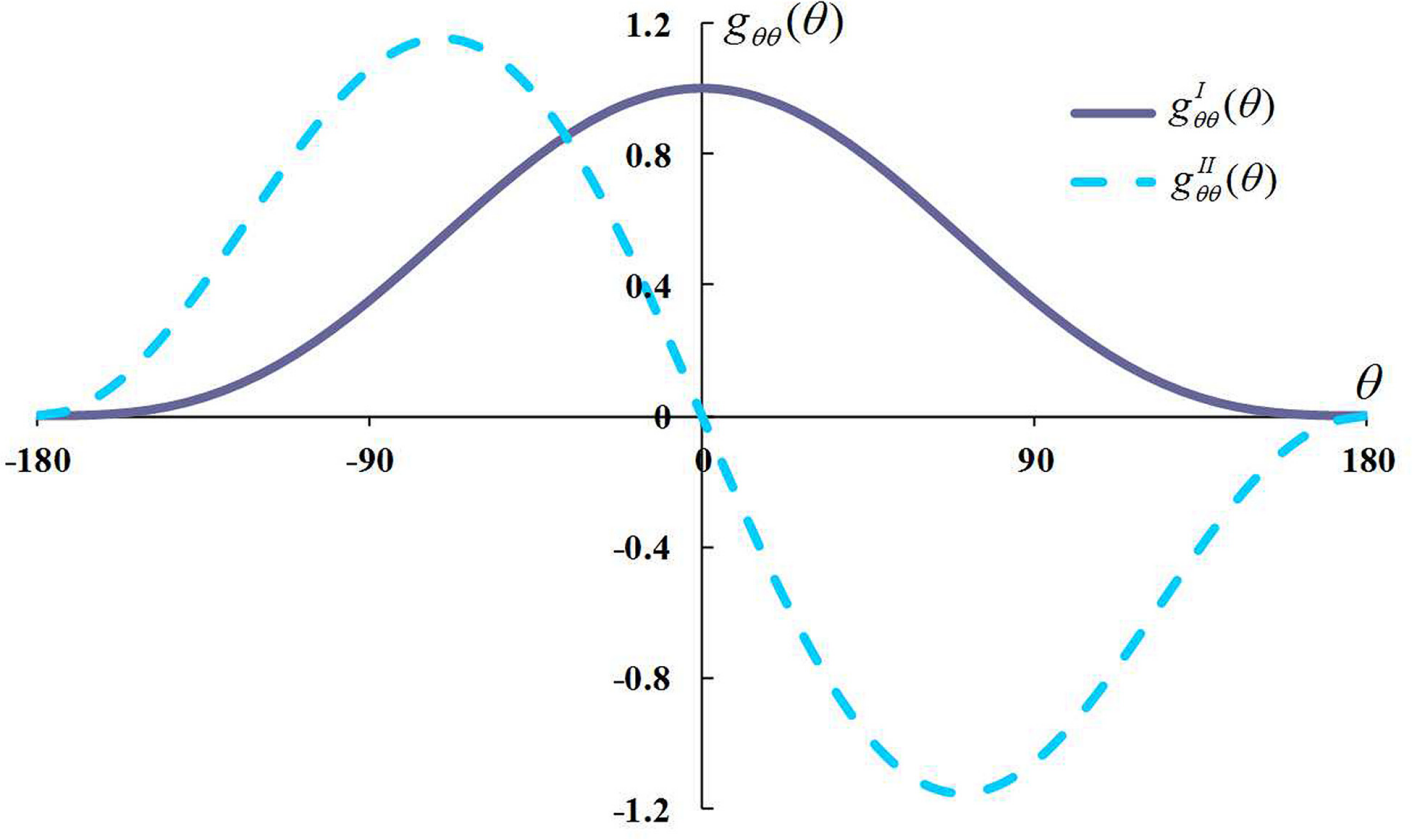
Stress analysis of the ellipse natural fracture.

Considering a vertical buried natural fracture in infinite elastic rock. We assume that the long axis of the elliptical cross section is 2*a*, the natural fracture is in a state of two-axial compression stress, and the surface produces a shear stress *τ*_*xy*_ under the combined stress field (maximum and minimum principal stress: *σ*_*xx*_ and *σ*_*yy*_). We construct a local rectangular coordinate system, *SON*, by using the center of the fracture surface as the origin and the *S* axis as the long axis. In the global coordinate system, *XOY*, the angle between the normal direction of the crack surface and the *ox* axis is *α* (as shown in Fig 7).

According to the 2-D linear elastic theory of fracture mechanics, natural fractures are an independent system unit. Therefore, when hydraulic fractures interact with natural fractures in the direction perpendicular to the normal stress caused by the combined stress field, the pressure component of the fracturing fluid can be expressed as the following:

$$p_{net}=\frac{1}{2}(1-\text{cos}\;2\alpha )p_{net(i)}$$ (18)

where *p*_*net*(*i*)_ is the net pressure inside the fracture at the intersection point between the natural fracture and hydraulic fracture, expressed in MPa.

Therefore, under the multi-fracture combined stress field, the type I SIF at the branch fracture tips that is produced by the normal stress can be defined as follows:

$$K_{I}=\underset{r\to 0}{\text{lim}}{\sqrt{2\pi r}\;p_{net}|}_{\alpha =0}=p_{net}\;\sqrt{\pi a}=\frac{1}{2}(1-\text{cos}\;2\alpha )p_{net(i)}\;\sqrt{\pi a}$$ (19)

The shear stress parallel to the natural fracture surface under the stress field can be obtained as follows:

$$\tau_{xy}=\sigma_{xx}\;\text{sin}\;\alpha \;\text{cos}\;\alpha -\sigma_{yy}\;\text{cos}\;\alpha \;\text{sin}\;\alpha =\frac{1}{2}(\sigma_{xx}-\sigma_{yy})\;\text{sin}\;2\alpha$$ (20)

Consequently, under a multi-fracture combined stress field, the type II SIF at the natural fracture tips produced by the shear stress can be defined as follows:

$$K_{II}=\underset{r\to 0}{\text{lim}}{\sqrt{2\pi r}\;\tau_{xy}|}_{\alpha =0}=\frac{1}{2}\text{sin}\;2\alpha (\sigma_{xx}-\sigma_{yy})\sqrt{\pi a}$$ (21)

## Results, Analysis and Discussion

We use the basic parameters in Table 1 to analyze the mechanism of fracture network propagation with SRV fracturing in tight oil reservoirs, including the variation rules of stress field formation, FPP, branch-fracture propagation angle and pressure.

**Table 1:** Basic parameters used for the results analysis and discussion.

Geological parameters	Data	Engineering parameters	Data
Poisson's ratio	0.25	Scope of the study area, m	10×10
Young's modulus, GPa	5	Hydraulic fracture half-length, m	2、5
Rock tensile strength, MPa	3.6、4.2	Fracture element length, m	0.2
Rock fracture toughness, MPa m^0.5^	0.8、1.6	Contact angle, degree	-90~90
Natural fracture half-length, m	0~10	Propagation net pressure, MPa	2、5
X direction normal stress, MPa	20	Net pressure on fracture surface, MPa	5
Y direction normal stress, MPa	10、15、20		

In this table, geological parameters are uncontrollable factors, engineering parameters are controllable factors.

### 4.1 Model Comparison and Validation

To compare the superiority of different model solution methods, a line fracture of length 6*a* subjected to a pressure *p* was analyzed. The analytical solution of the normal discontinuous displacement can be given by the following:

$$D_{n}(x)=\frac{12\;pa(1-\nu^{2})}{E}\sqrt{1-\frac{x^{2}}{9a^{2}}}$$ (22)

We divide the line fracture into three equal elements, each of which has a length of 2*a*. Considering the solutions of analytical method, DDM and modified DDM, the calculated results including the normal discontinuous displacement and normal stress of fracture in the N direction can be obtained (Table 2).

**Table 2:** Comparison of calculation results under different model solution methods.

Solution methods	Element 1			Element 2			Element 3		
	*M*	*σ*_*nn*_	*RE*, %	*M*	*σ*_*nn*_	*RE*, %	*M*	*σ*_*nn*_	*RE*, %
Analytical solution	8.94	0.95	/	12.00	3.82	/	8.94	0.95	/
DDM’s solution	11.78	1.25	31.67	14.14	4.50	17.33	11.78	1.25	31.67
Modified DDM’s solution	9.34	0.99	4.47	12.57	4.00	4.75	9.34	0.99	4.47

In this table, $M=-\frac{D_{n}E}{pa(1-\nu^{2})}$; *RE* is the relative error; *D*_*n*_ is the normal discontinuous displacement; *E* is the young's modulus; *p* is the net pressure; *v* is the poisson's ratio; *a* is the half length of element; *σ*_*nn*_ is the normal stress of fracture in the N direction.

As shown in Table 2, for the normal discontinuous displacement and normal stress at elements adjacent to the fracture center and tips, the modified DDM’s solution is in excellent agreement with the analytical solutions and more accurate than the DDM’s solution. The relative errors of modified DDM’s solutions are always less than 5% for all the elements. But the relative errors of DDM’s solutions are 17.33% and 31.67% for the center element and both sides of the elements, respectively. Therefore, the simple DDM’s solution cannot give reliable results for field points close to the fracture tip. The more elaborate DDM based on the Lagrange interpolation (modified DDM) will be necessary in this case.

### 4.2 Variation Rule of Ground Stress Field

**Fig 8:**
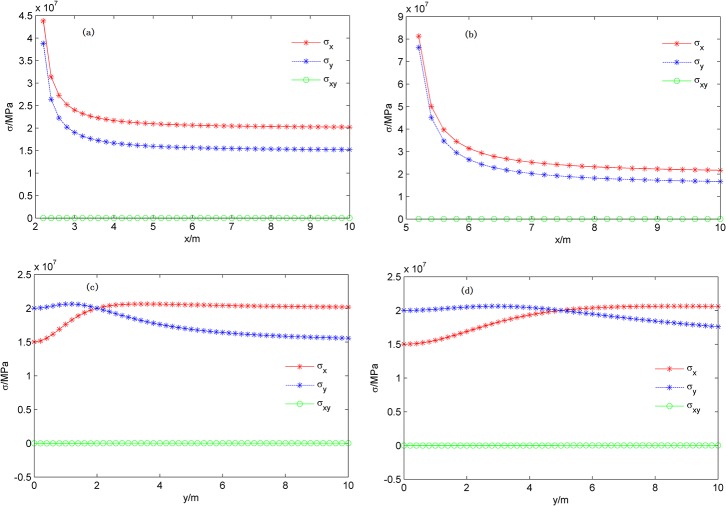
Stress distribution curves on the X and Y axis over different fracture lengths. (a) Stress distribution on the X axis (L_**f**_=2 m); (b) Stress distribution on the X axis (L_**f**_=5 m); (c) Stress distribution on the Y axis (L_**f**_=2 m); (d) Stress distribution on the Y axis (L_**f**_=5 m).

**Fig 9:**
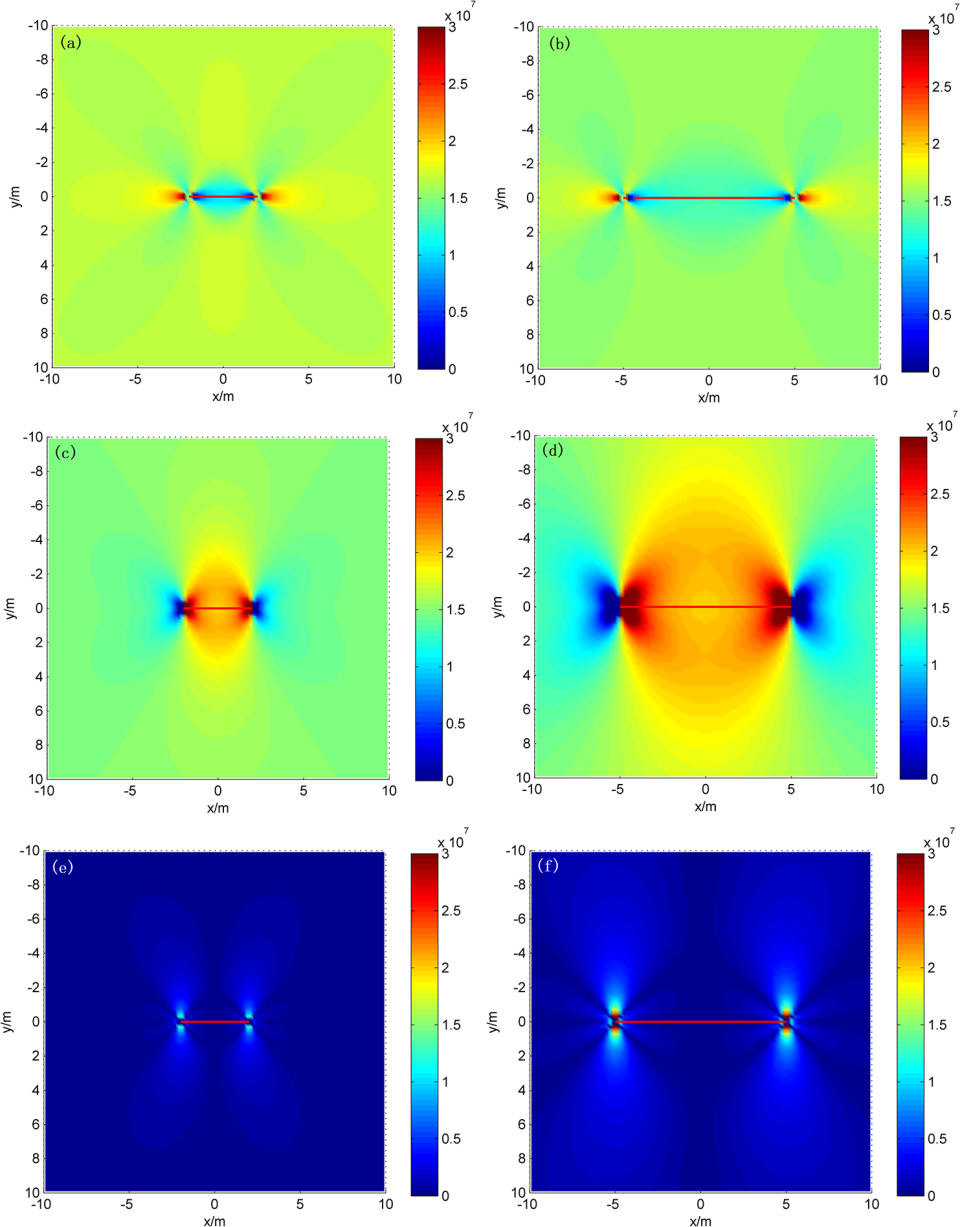
2-D ground stress field distribution over different fracture lengths. (a) Normal stress in the X-direction (L_**f**_=2 m); (b) Normal stress in the X-direction (L_**f**_=5 m); (c) Normal stress in the Y-direction (L_**f**_=2 m); (d) Normal stress in the Y-direction (L_**f**_=5 m); (e) Shear stress (L_**f**_=2 m); (f) Shear stress (L_**f**_=5 m)

According to the formula for the combined stress field, the stress distribution curve on the X and Y axis and the 2-D ground stress field distribution over different fracture lengths can be obtained, the results are shown in Fig 8 and Fig 9.

Fig 8A–8D and Fig 9A–9F compare the differences of the stress distribution curves on the X and Y axis and the stress field distribution over different fracture lengths during a signal fracture propagation.

1. The phenomenon of local stress concentration at fracture tips is induced by the stress near the fracture tips, which changes dramatically in the X-direction. The normal stress in the X-direction increases at the fracture tips and declines rapidly far away from the fracture tip; the normal stress in the X-direction is always greater than the Y-direction.
2. The initial orientation of the maximum horizontal principal stress deflects in the vicinity of the fracture in the Y-direction. The normal stress in the X-direction is less than in the Y-direction for the deflection area and is consistent with the initial orientation out of the deflection area.
3. In the process of fracture propagation, the induced shear stress is small and forms the approximate shape the numeral "8" in the 2-D stress field distribution.

During fracture propagation, the stress influence range increases gradually, the phenomena of local stress concentration and stress deflection quickly become increasingly evident, and the stress field changes intensify.

### 4.3 Mechanical Analysis of the FPP

**Fig 10:**
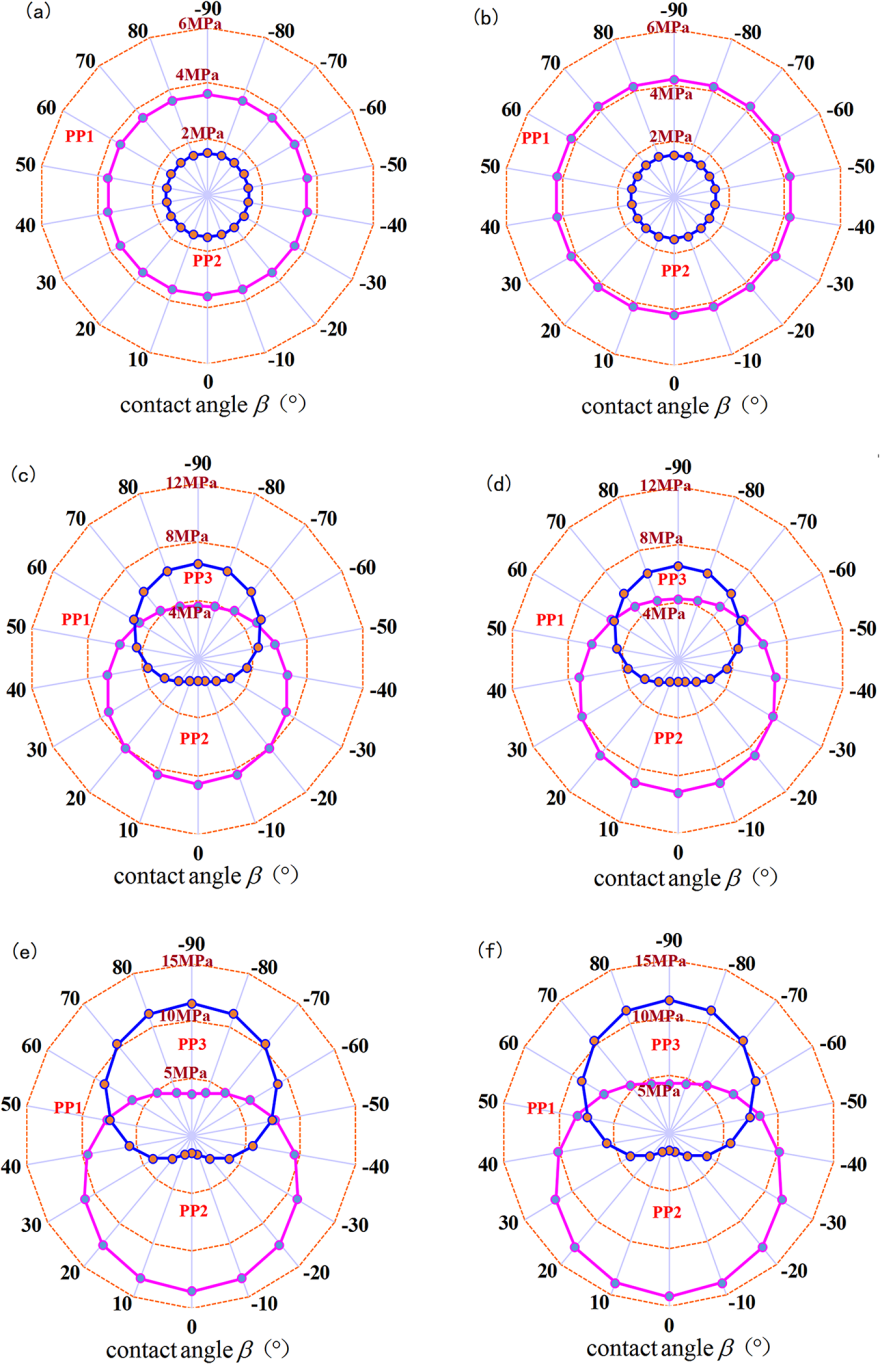
Mechanical analysis for FPP under different control conditions. (a) Δσ=0 MPa, T_**0**_=3.6 MPa; (b) Δσ=0 MPa, T_**0**_=4.2 MPa; (c) Δσ=5 MPa, T_**0**_=3.6 MPa; (d) Δσ=5 MPa, T_**0**_=4.2 MPa; (e) Δσ=10 MPa, T_**0**_=3.6 MPa; (f) Δσ=10 MPa, T_**0**_=4.2 MPa.

FPP 3 forms a fracture network system that combines the main fracture and branch fractures whereas FPP 1 can only form the fracture network that has a specific bandwidth. Therefore, the mechanical conditions of FPP 3 are the most favorable for forming a complex fracture network, followed by FPP 1; FPP 2 only forms a single fracture. Considering the mechanical formula for the FPP and the factors of the contact angle, horizontal stress difference and net pressure inside the fracture, three FPPs can be obtained under different mechanical control conditions, as shown in Fig 10.

Fig 10A-10F compare the differences of FPP under different control conditions. The contact angle, stress difference and net pressure inside the fracture and other factors combined determine the FPP.

1. The stress difference has a greater effect on FPP. When the stress difference is 0 MPa, only the FPP 1 and FPP 3 conditions may arise; whereas when stress differences exist, all three FPPs may appear. For greater stress differences, greater mechanical values are required to produce FPP 3, which is not conducive to the formation of a fracture network.
2. The contact angle has some influence on FPP. A small contact angle with increases in pressure during fracture treatment will cause the intersection between the hydraulic fracture and natural fracture to first satisfy the mechanical conditions of FPP 1. Then, this condition will divert the natural fracture propagation to cause FPP 3 by passing through natural fractures, forming a more complex fracture network. A greater contact angle corresponds to a higher critical net pressure required to form the fracture network, i.e., resistance to the formation of the complex fracture network.
3. The rock tensile strength influences the hydraulic fractures passing through the natural fracture and directly affects the boundary between FPP 1 and FPP 3. Greater rock tensile strength corresponds to a greater value of the critical net pressure required to generate FPP 3, which is required for SRV fracture treatment.

### 4.4 Variation Rule of Branch Fracture Propagation Angle

**Fig 11:**
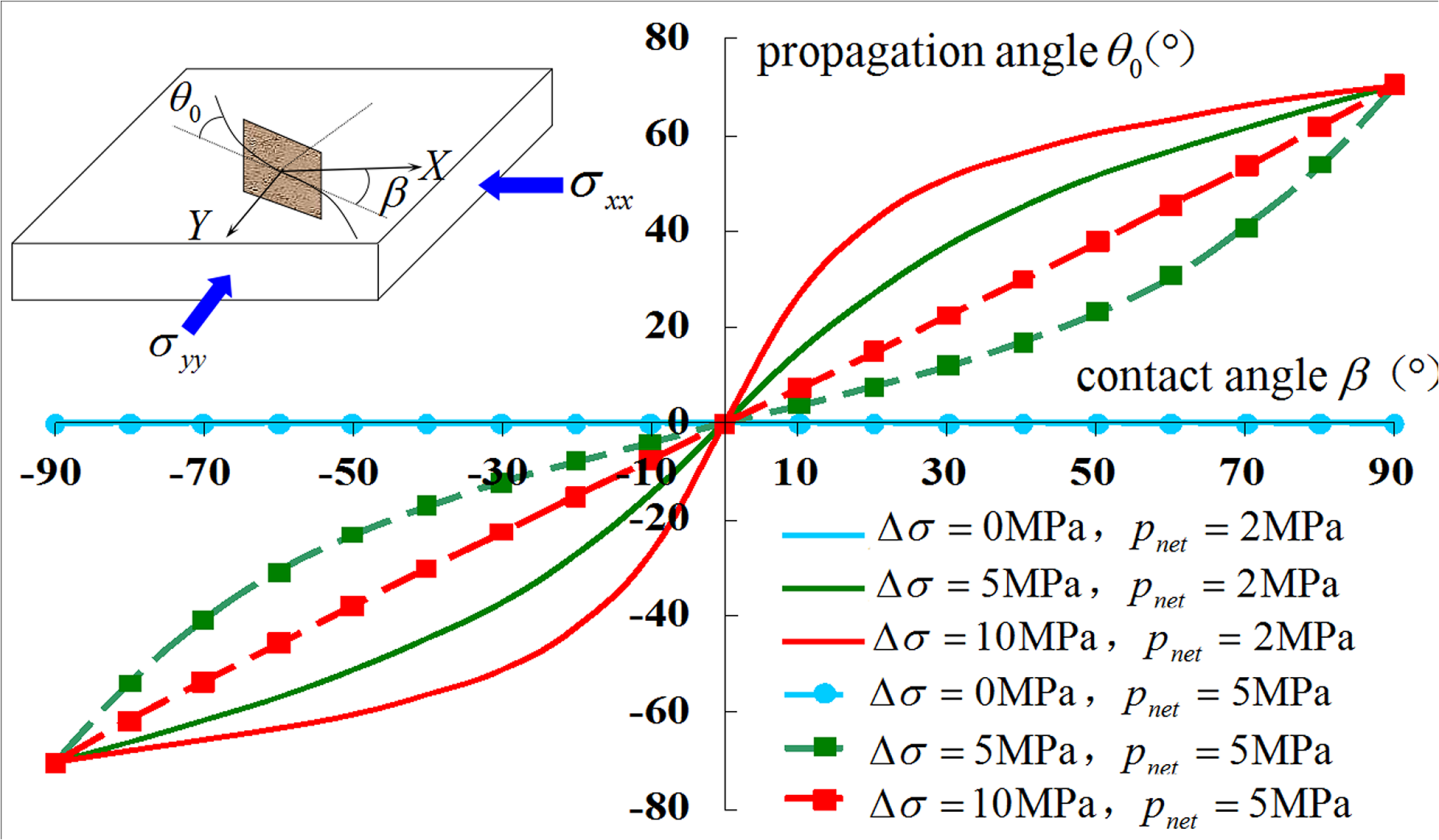
Variation curve of branch fracture propagation angle.

In light of the formula for the compound fracture propagation angle, the variation curve of the branch fracture propagation angle with contact angle, the horizontal stress difference and net pressure inside the fracture can be calculated, and the result is shown in Fig 11.

Fig 11 shows that when the hydraulic fracture interacts with the natural fracture, the contact angle, net pressure inside the fracture and stress difference have a decisive effect on the trajectory of the branch fracture propagation, with multiple effects:

1. The contact angle is an uncontrollable parameter. When the hydraulic fracture interacts with the natural fracture along the direction of maximum principal stress in a tight reservoir, the branch fracture propagation angle increases with the increasing contact angle. When the contact angle is 0°, the rock tensile failure will cause a pure type I (open type) fracture, in which the hydraulic fracture passes through the natural fracture without deflection. When the contact angle is 90°, shear and slip failure occur, causing pure type II (slip type) fractures; the deflection angle of the branch fracture then reaches a maximum value of 70.53°.
2. Greater net pressure favors the formation of a complex fracture network. When the stress difference is constant and the net pressure inside the fracture increases, the branch fracture propagation deflection angle decreases. Additionally, the radius of curvature of the deflecting propagation trajectory increases. Therefore, the branch fracture can encounter more natural fractures in the vertical direction, increasing its bandwidth. This process is conducive to the production of a more complex fracture network. During hydraulic fracturing, the net pressure is dynamic with fracture propagation and is a controllable factor; thus, the net pressure can be increased by increasing the flow rate of the fracturing fluid in a tight oil reservoir.
3. Smaller stress differences favor the formation of the complex fracture network. When the net pressure inside the fracture is constant and the stress difference increases, on the one hand, the branch fracture deflecting propagation angle increases and the radius of curvature of the deflecting propagation trajectory decreases. Additionally, the branch fracture converges towards the direction of principal stress over a small range because of the higher pressure drop within the fracture, allowing the branch fracture to continue to propagate more difficultly. On the other hand, when the stress difference are too low, the hydraulic fracture propagates more easily along the natural fracture surface that has a weak strength. This situation is not conducive to increasing of band length of the fracture network because it reduces the chance to encounter more natural fractures in the horizontal direction.

Therefore, the SRV fracture treatment requires not only the hydraulic fracture to propagate farther along the direction of maximum principal stress to encounter more natural fractures but also a smaller propagation angle to produce a larger fracture-network band width and to encounter more natural fractures. Combining both of these conditions, we can achieve the formation of a complex-fracture network system.

### 4.5 Variation Rule of Branch Fracture Propagation Pressure

**Fig 12:**
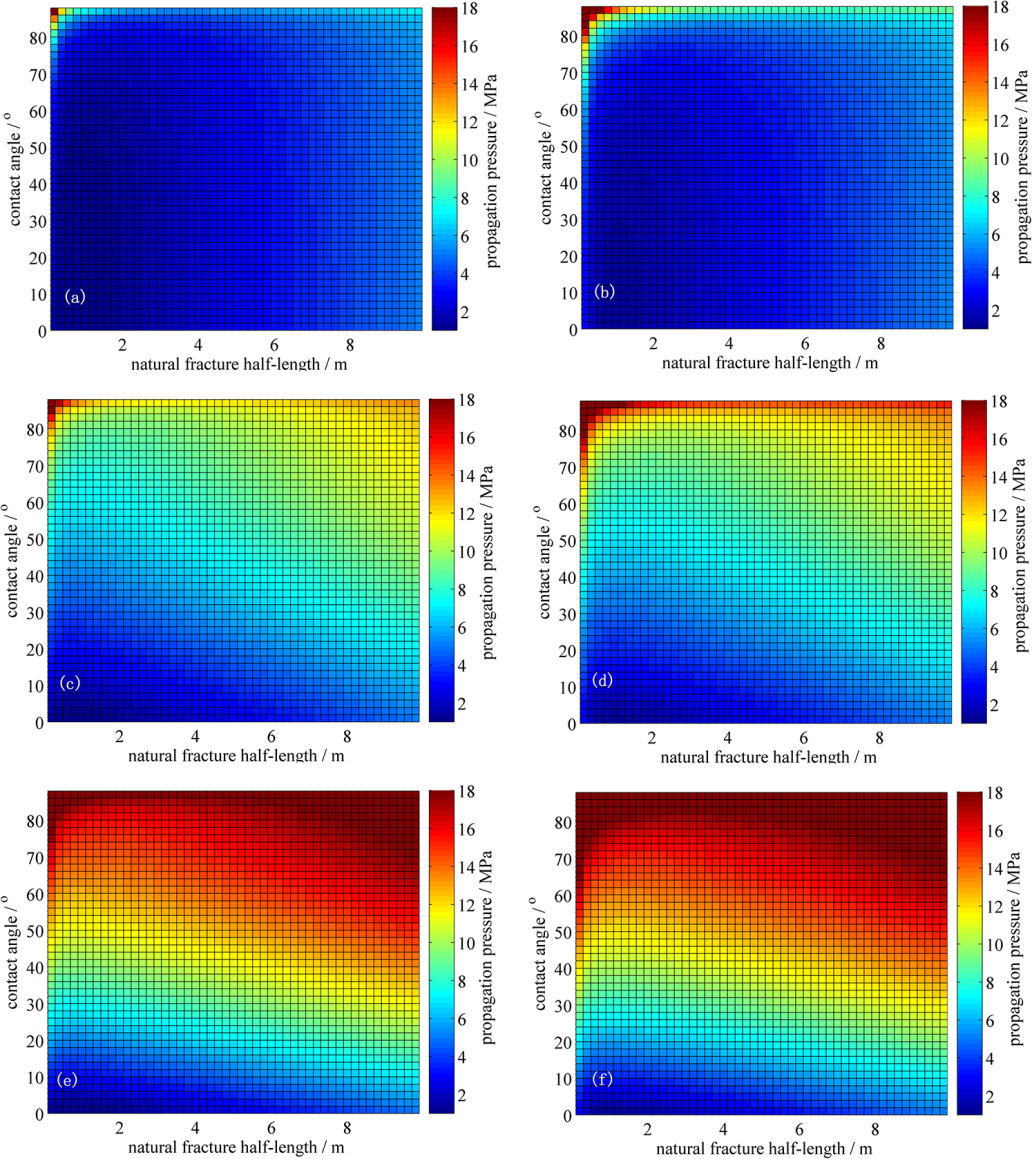
Variation rule of branch fracture propagation pressure. (a) Δσ=0 MPa, K_**IC**_=0.8 MPa m^0.5^; (b) Δσ=0 MPa, K_**IC**_=1.6 MPa m^0.5^; (c) Δσ=5 MPa, K_**IC**_=0.8 MPa m^0.5^; (d) Δσ=5 MPa, K_**IC**_=1.6 MPa m^0.5^; (e) Δσ=10 MPa, K_**IC**_=0.8 MPa m^0.5^; (f) Δσ=10 MPa, K_**IC**_=1.6 MPa m^0.5^.

Based on the fracture initiation and propagation criteria of mixed-mode I and II, the variation rule of branch fracture propagation pressure with contact angle, natural fracture half-length, horizontal stress difference and rock fracture toughness can be calculated, as seen in Fig 12.

Fig 12A-12F compares the differences in branch fracture propagation pressure under different control conditions, of which the following are several of the notable effects:

1. The contact angle has a large effect on the propagation pressure required to divert the propagation of the natural fracture. When the contact angle is 0°, pure type I open fractures are produced. These fractures are produced simply by overcoming the minimum principal stress and rock fracture toughness. The path of the hydraulic fracture coincides with the natural fracture and passes through it. When the contact angle increases further, the effective net pressure at the intersection of the hydraulic fracture and natural fracture decreases, and the required propagation pressure required, hindering the formation of a fracture network. However, when the contact angle approaches 90°, pure type II fractures occur, in which steering the natural fracture requires maximum net pressure. Therefore, producing a fracture network is difficult.
2. The natural fracture half-length has some influence on the propagation pressure required to induce a branch fracture. On the one hand, with increases in the natural fracture length, the generated equivalent-stress intensity factor increases, reducing the magnitude of the net pressure that can induce the extension of a branch fracture; this reduction in the magnitude of net pressure occurs when the natural fracture is shorter. On the other hand, longer natural fractures correspond to a greater loss of net pressure at the tip when the hydraulic fractures encounter the natural fractures, resulting in the need for higher pressure to form a fracture network.
3. Stress differences have a greater effect on the propagation pressure required for the deviation of the natural fracture. When the stress difference increases, the net pressured required to steer the natural fractures to extend along the branch increases markedly. It becomes more difficult to expand or initiate a branch; this difficulty occurs because of the increases in the branch propagation angle. The increased angle reduces the radius of curvature to a smaller trajectory that reaches the main branch in the direction of the maximum principle horizontal stress. This intersection decreases the net pressure rapidly, resulting in the need for a higher pressure to induce a branch fracture.
4. The rock fracture toughness has a smaller effect on the propagation pressure required for the deviation of the natural fracture. With increases in the rock fracture toughness, the natural fracture propagation resistance increases. Therefore, natural fractures require greater pressure to produce a net branch fracture, resulting in increased difficulty to form a fracture network.

The simulation results show that the natural fracture not only steering extends with the in-situ differential stress and rock fracture toughness but is also influenced by the length and orientation of the natural fractures. Therefore, during the SRV fracturing in the tight oil reservoir, more mature and dense natural fractures are suggested to be selected in the block, and the influence of the natural fracture length and orientation of the fracture network morphology should be considered.

### 4.6 Influence of SRV on Oil Well Performance

**Fig 13:**
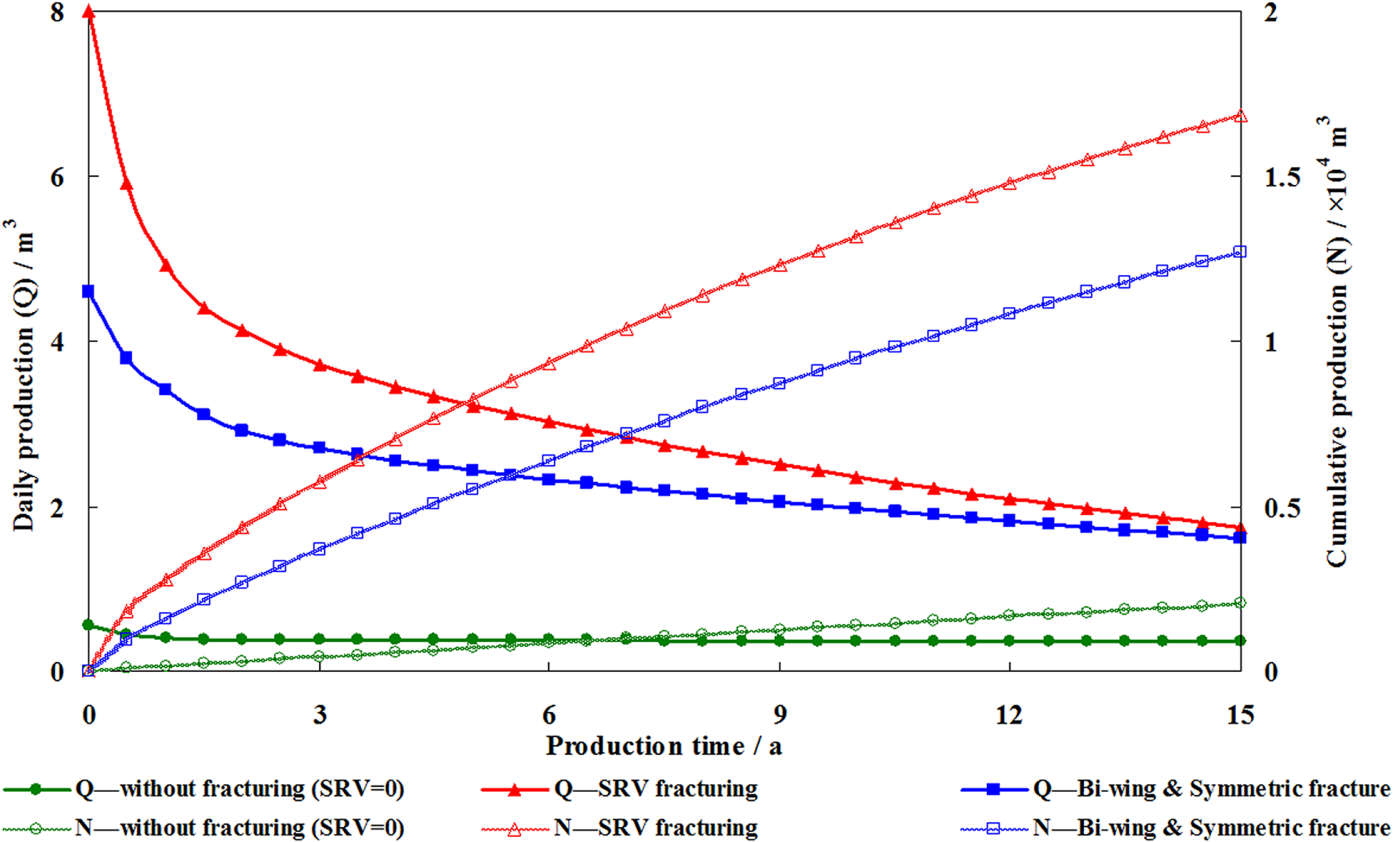
Production performance of oil well under different fracturing patterns.

Varied lithologic compaction, poor physical property and high flow resistance are inherent characteristic for tight reservoir. Hydraulic fracturing technology are widely used to change the flowing regime near the wellbore, and increase the channel of oil flowing to improve the well performance. To reveal the influence of SRV on oil well performance, considering the situation of without fracturing (SRV=0), conventional fracturing (Bi-wing & Symmetric fracture) and SRV fracturing, the production performance including daily production and cumulative production curve of oil well can be obtained by numerical reservoir simulator, as shown in Fig 13.

Results show that without fracturing, oil production is extremely low in tight oil reservoir and it’s hard to achieve economic rates, so hydraulic fracturing is a necessary process to accelerate production. Conventional fracturing could create bi-wing & symmetric fracture, providing significantly more fracture-surface contact with the reservoir, thus accerates oil production obvisously. However, SRV fracturing produces higher rates with the following mechanism.

SRV fracturing could communicate hydraulic fracture with natural fracture and create 3D fracture network with certain length, width and height, that will increase the stimulated reservoir volume and enhance the overall system permeability. In tight oil reservoir, diffusivity-related pore-pressure changes cannot move very far from the fracture, so little fluid is produced outside the extent of the SRV. Within the SRV, SRV maximize the contact area between fracture-surface and matrix, so oil could flow from the matrix to the created fracture network through the shortest path easily. SRV could maximize producing ratio and recovery percent of reservoir, having significant effect on oilfield production.

So it’s necessary to study the mechanism of fracture network propagation with SRV fracturing in tight oil reservoirs. The relevant results studied including geological conditions and engineering treatments have both theoretical significance and practical application value by contributing to a better understanding of fracture network propagation mechanisms in tight oil reservoirs and to the improvement of the science and design efficiency of reservoir fracturing.

## Conclusions

SRV fracturing has emerged as an important technology to exploit oil/gas from unconventional reservoirs, in which fluids are injected at high rate and pressure to crack the reservoir rock in order to create pathways with higher permeability to accelerate production. This paper analyzes the mechanism of fracture network propagation with SRV fracturing in tight oil reservoirs from both geological and engineering perspectives, the following conclusions can be drawn:

1. During the process of fracture propagation, the stress field produces local stress concentration at the fracture tips, and the initial orientation of maximum horizontal principal stress deflects into the area around the fracture.
2. Propagation pattern 3 (in which a hydraulic fracture passes through a natural fracture, thereby inducing the diversion of the natural fracture propagation) is the most conducive to forming a network fracture system by combining a principle fracture and a branch fracture.
3. A specific set of reservoir geological attributes (higher brittle mineral contents and stronger elastic characteristic of the rock (Poisson's ratio and Young's modulus), lower tensile strength and fracture toughness of rock, mature natural fractures (natural fracture density), and lower stress anisotropy (horizontal stress difference)) are more conducive to the propagation of hydraulic fractures into a complex fracture network. According to the engineering conditions of fracturing operations, higher net treatment pressure (propagation pressure), reasonable fracturing azimuth direction (contact angle) and larger fracturing scale (hydraulic fracture length) will assist the formation of complex fracture networks with SRV fracturing in tight oil reservoirs.
